# Family Communication and Verbal Child-to-Parent Violence among Adolescents: The Mediating Role of Perceived Stress

**DOI:** 10.3390/ijerph16224538

**Published:** 2019-11-16

**Authors:** Teresa Isabel Jiménez, Estefanía Estévez, Coral M. Velilla, José Martín-Albo, María Luisa Martínez

**Affiliations:** 1 Department of Psychology & Sociology, University of Zaragoza, Ciudad Escolar s/n, 44003 Teruel, Spain; tijimgut@unizar.es (T.I.J.); coral_marin@hotmail.com (C.M.V.); jmartina@unizar.es (J.M.-A.); 2 Departament of Health Psychology, University of Miguel Hernández, Avda. de la Universidad s/n, Edificio Altamira, 03202 Alicante, Spain; 3 Enforcement Center for Judicial Measures, Partida de Bacarot, Pol. B, 97 bajo, 03114 Alicante, Spain; mlmartinezpastor@hotmail.com

**Keywords:** adolescents, verbal child-to-parent violence, perceived stress, family communication

## Abstract

In recent years, there has been an increase in the number of reported incidents of child-to-parent violence (CPV); however, this type of intra-family violence remains vastly understudied compared with other forms of family violence. The aim of this study is to analyze the relationship between family communication and verbal CPV through the mediation of adolescent perceived stress. The sample consisted of 2399 Spanish students of both genders between the ages of 11 and 20 years. Results show that problematic family communication is a risk factor for the presence of verbally abusive behavior towards parents, with a direct and indirect relationship through perceived stress. Open family communication is presented as a protective factor against verbally abusive behavior due to a negative relationship with perceived stress. Results point to a mediating role of perceived stress, which would explain the mechanism which links the quality of family communication to verbal violence towards parents. Implications of these results are discussed.

## 1. Introduction

In recent years, there has been a sharp increase in the number of reported incidents of a, to date, little studied type of intra-family violence, such as child-to-parent violence (CPV) [1,2,3]. The office of the state prosecutors of Spain declared in its 2009 annual report that CPV was the most worrying crime committed by adolescents due to its prevalence and incidence. The 2016 version of this report indicates that, with regards to crime in minors, there has been a decline in all types of delinquency over recent years, with the exception of family violence against parents [2].

CPV or parent abuse is defined as any act committed by children that makes parents feel threatened, intimidated, and controlled [4]. The various proposed definitions of CPV to date agree with the presence of reiterated aggressive behaviors of children against their parents. These can take the form of physical (e.g., pushing, blows), verbal (e.g., insults, threats), and other non-verbal violence (e.g., blackmail, economic infractions) [5]). This study focuses on verbal violence due to its greater frequency and early onset. Indeed, in interviews with 20 families who had requested help with this type of problem, Eckstein [6] found that the first type of violence to appear was verbal, with physical actions appearing later in a progression and escalation of abuse episodes. A diverse range of studies have supported that psychological aggressions are more common than physical ones and that verbal abuse is a predictor of physical abuse [7,8].

This research lies within the framework of the ecological systems theory of human development [9,10] and, in particular, within the family circumplex model [11,12]. From the first, violence is understood as a relational phenomenon in which a range of variables interact. These vary in level from individual variables to macro-social ones. Therefore, the analysis of CPV needs take into account the progressive mutual adaptation of the characteristics of the developing child and the characteristics of his or her immediate surroundings. Cottrell and Monk [13] have revealed a number of interacting factors that contribute to CPV, and these occur across psychological, intrafamilial, social, and political spheres. In this study, we focus on the family context, making its interaction with psychological characteristics of the developing child a key object of analysis.

Among the multiple components of the family context, the communication between its members is considered a good indicator of how well the family system functions [14]. According to the circumplex model of the marital and family system [11,12], family communication is what makes the emotional linking of family members possible while also allowing a certain flexibility in structure, roles, and rules [15]. Open communication between family members (positive communication based on the free exchange of information, understanding, and the satisfaction with the relationships) facilitates the adaptive resolution of family transitions as adolescence, whereas problematic communication (overly critical or negative, focused on a resistance to sharing information and affection) obstructs family development. In this way, the presence of problems in family communication is considered a reliable indicator of family dysfunction [11,16]. Previous studies reveal the close link between the quality of family communication and behavioral problems in children. Open communication has been related to better behavioral and psychological adjustment of adolescent children [17,18], while problems in family communication has also been linked to children’s disruptive behavior [19], violent behavior [20,21], delinquent behavior [22], and substance abuse [23].

In relation to CPV, some studies have indicated that open communication is a protective factor against CPV [24,25]. Studies have also found that a shortfall in family communication is related with adolescent violent behavior toward their parents [24,25]. Specifically, Pagani et al. [8,26] suggest that, in cases of CPV, problematic interactions exist between parents and children which generally arise throughout childhood. The problems that these authors outline are that children and parents do not share many activities in their everyday life and that there is scant positive communication between them. In a descriptive study of CPV cases, Tobeña [27] concludes that parents, professionals, and adolescents report perceiving a low level of communication in the family. In summary, the presence of problematic communication in families is related to the occurrence of CPV and can thus be considered a risk factor for the development of these behaviors. However, these studies do not outline the mechanisms which link problematic communication to CPV.

Stress perceived by adolescents (the extent to which they appraise that their demands exceed their ability to cope) is a potential mediating variable to consider in this relationship, given its consistent link with violent behavior in adolescents [28,29,30] and, more specifically, with violence toward parents [13,24,25,31,32,33,34,35]. These studies put forward the idea that when adolescents perceive elevated levels of stress, they can react with violence to the environmental demands. Concerning the role of the family context in the stress perceived by the adolescents, Llamazares et al. [36] indicate that certain characteristics of the family situation, such as communication problems, constitute some of the main sources of stress in adolescents. In a similar vein, Herrero et al. [37] found that open communication in families was related with less perceived stress in adolescent children, while family communication problems were related with increased symptoms of stress. In light of this, perceived stress by adolescents seems to be closely linked to quality indicators of family functioning as family communication. However, the relation between both variables has not been examined in the context of CPV.

Instead, most of the studies either focus their analysis on individual factors or are centered around family characteristics as explanatory contextual elements of CPV [38]. From an ecosystemic approach to development, it is necessary to jointly analyze the influence of these two areas due to the consideration of the close relationship between the individual characteristics of the adolescent and the characteristics of the environment in which he or she develops [10]. From this point of view, adolescent self-perceptions are closely related to family functioning, the latter deriving from its role as the relational context closest to the individual’s development. Accordingly, in the study of CPV, there is a need to identify third variables which may help to clarify the linking mechanism between family variables and CPV. This brings us to the main objective of this study: The proposal of a descriptive model of the relationship between family communication, perceived stress, and verbal CPV. Based on the research revised previously, we hypothesize that open family communication will be negatively related to perceived stress and verbal CPV, while problematic family communication will be positively related with these two variables. We also predict that perceived stress will have a mediating role between family communication and CPV.

## 2. Materials and Methods

### 2.1. Participants

A total of 2399 Spanish adolescents of both sexes (50% male, 50% female) took part in this study. The students, aged from 11 to 20 years old (M = 14.66; SD = 1.812), came from four schools in the regions of Valencia and Andalusia. In each region, participants were selected using a stratified cluster sampling method [39]. It is assumed that there is a sample error of ±2.3%, a 95% confidence interval, and a population variance of 0.50. The sampling units were schools, public and subsidized, picked from a list of schools in rural and urban areas. All the students of compulsory secondary education and baccalaureate within each school participated in the survey. A series of prior analyses of differences of means were conducted on the target variables of the study as a function of the location of the school and its public or subsidized condition, without finding any statistically significant differences.

### 2.2. Procedure

Firstly, a letter was sent to the selected schools explaining the research project. The school principal was subsequently contacted by telephone and the detail of the project was explained. Consent forms were sent to the parents, along with a letter from the principal explaining the nature of the research. After obtaining the relevant permissions, a seminar was held with the teaching staff of each school to explain the objectives and scope of the study. The research was carried out by a group of trained and experienced researchers to provide students with the necessary support to successfully complete the questionnaires. We explained the goals of the study to the students, informed them that participation was voluntary and anonymous, and required their consent. Participants filled out the scales in their usual classrooms during a regular classroom period. The order of administration of the instruments was counterbalanced in each class and in each school. The ethics committee of the hosting university (University of Miguel Hernández) granted ethical approval. The study met the ethical values required for research on human beings, respecting the basic principles included in the Helsinki Declaration.

### 2.3. Measures

#### 2.3.1. Degree of Openness and Extent of Problems in Family Communication

We used the parent-adolescent communication scale (PACS) [40], adapted to Spanish by Musitu et al. [41]. The questionnaire is divided into two subscales, one referring to communication with the mother and the other referring to communication with the father. Both consist of 20 items and contain two sub-scales which measure the degree of openness (e.g., “I usually believe what he tells me.”) and the extent of problems in family communication (e.g., “They say things to me that hurt me.”). Responses range from 1 (never) to 5 (always). In our data, McDonald’s [42] omega reliability for the open communication and communication problems sub-scales were 0.96 and 0.83, respectively.

#### 2.3.2. Perceived Stress

We used the Spanish version [43] of the perceived stress scale (PSS4) [44]. The PSS is a 4-item scale which measures the degree to which respondents appraise situations within the last month as stressful (e.g., “I felt I was unable to control the most important things in my life”). Items are rated on a scale of 5 points from 0 (never) to 4 (very often). In the present study, McDonald’s omega reliability for this scale was 0.81.

#### 2.3.3. Child-to-Parent Verbal Violence

The child version of conflict tactics scales (CTS2) by Straus and Douglas [45] was used and adapted to Spanish by Gámez-Guadix and Calvete [46]. The three items of verbal violence (e.g., “I shout or have shouted at my parents.”), referring equally to the father and the mother, were used in this study. The participants report on the frequency with which they have verbally abused their parents on a 5-point scale (from never to many times). In the present study, McDonald’s omega reliability for this scale was 0.95.

### 2.4. Data Analysis

The statistical program SPSS Amos v.19 (Amos Development Corporation, Chicago, IL, USA) was used. Missing values were calculated using the linear interpolation method. Firstly, the descriptive statistics of the observable variables (mean, standard deviation, skewness, and kurtosis) and the bivariate correlations between them were calculated. Secondly, a structural equation model was then used to analyze the relationships between the variables using a two-step process, as recommended by Anderson and Gerbing [47]. The measurement model was analyzed to check if each latent construct was measured through its indicators. Next, the structural model was calculated in order to analyze the relationships between family communication (degree of openness and extent of problems), perceived stress, and verbal child-to-parent violence. In order to evaluate each of the models, the covariance matrix and a combination of adjustment indices, both absolute and relative, were used: The comparative fit index (CFI), the incremental fit index (IFI), the root mean square error of approximation (RMSEA), and the standardized root mean square residual (SRMSR). For the SRMSR, values below 0.08 are indicative of a good model fit. For the RMSEA, values below 0.06 are considered indicative of a good fit, below 0.08 of a fair fit, between 0.08 and 0.10 of a mediocre fit, and above 0.10 of a poor fit [48], and the CFI and IFI values above 0.90 indicate an acceptable fit for the model [49]. Finally, the sample was randomly sorted into two by the SPSS program to provide data to run a replica of the analysis. Confidence intervals were calculated using the bootstrap method with 500 samples.

## 3. Results

### 3.1. Descriptive Analysis

Table 1 shows the descriptive statistics for the studied variables. As we can see, the univariate skewness and kurtosis indices are lower than two. This indicates a similarity to a standard curve [50], except in items 2 and 3 of the verbal CPV. The analysis of bivariate correlation revealed significant correlations between all of the studied indicators except for item 3 of problematic communication, which only shows significant correlations with items 1 and 4 of the perceived stress scale and item 2 of the verbal CPV scale.

### 3.2. Analysis of Structural Equations

An analysis of structural equations was carried out to test the hypothesized relations between the variables. The tested model was identified as each latent variable had at least two indicators [51,52]. The specific latent variables used wereoppen communication (three indicators), communication problems (three indicators), perceived stress (four indicators), and verbal CPV (three indicators).

#### 3.2.1. Measurement Model

In order to confirm the measurement model, a confirmatory factorial analysis was carried out. An oblique model encompassing all of the latent variables of the theoretic structural model was used. Bearing in mind that the Mardia coefficient was high (61.42), the model fit was tested with a maximum likelihood method, together with standard bootstrapping with 500 resamples. This process offers an average of the estimations derived from the samples obtained from the bootstrap and their standard errors.

Furthermore, the bootstrapping process compares the values estimated without bootstrapping with the averages obtained from the bootstrap resampling in order to gauge the level of bias. The confidence intervals (differences between the highest and lowest of the estimated values resulting from the bootstrapping resampling) of the regression weightings and the standardized regression weightings indicate that the estimated values were considerably different from 0, bearing in mind that the lack of normality does not affect the estimations [53].

The results of the measurement model were acceptable under the proposed cut points, and the estimated parameters possessed significant values given that they did not include a 0 value. Specifically, the fit indices were: CFI = 0.92; IFI = 0.92; RMSEA = 0.08; SRMSR = 0.06; df = 59; X^2^/df = 7.92. All the parameters were significant (*p* < 0.01), and all latent variables correlated between them, with values between −0.40 (communication problems with perceived stress) and 0.61 (communication problems with verbal CPV).

#### 3.2.2. Structural Equation Model (SEM)

The structural equation model (SEM) calculated proposed that each of the two latent exogenous variables (open communication and communication problems) separately influenced the individual latent endogenous variable (perceived stress) and the verbal CPV. In addition, a covariation relation was observed between the two family latent exogenous variables. The individual latent endogenous variable in turn influenced the verbal CPV latent endogenous variable. The covariances and omegas obtained from the estimated parameter in the SEM are presented in Table 2.

**Figure 1:**
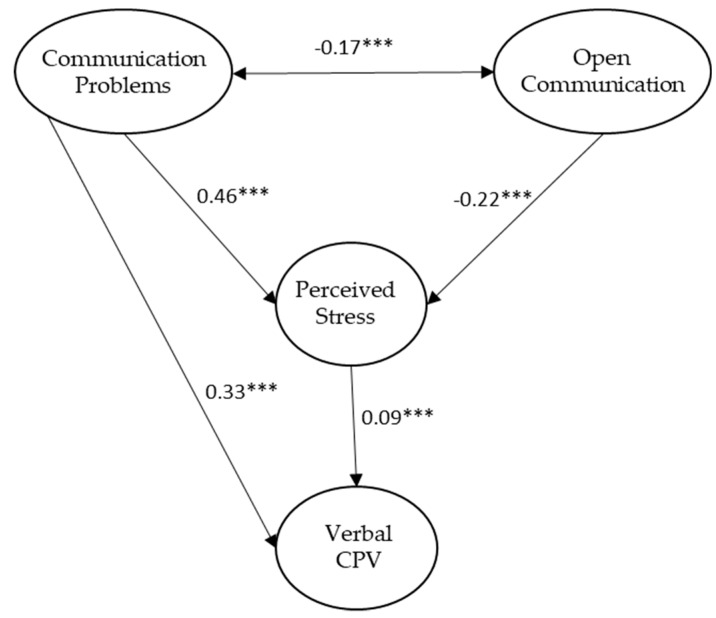
Standardized solution of the respecified structural model. Only estimates significant at *p* < 0.05 or less are provided. *p* < 0.001.

The results of the structural equation analysis showed an adequate goodness-of-fit, which is presented in Table 3 (see base model). According to the confidence intervals, all parameters were significant (*p* < 0.01); however, we found that open communication was not directly related with verbal CPV. This led to a respecification of the model, and the path open communication–verbal CPV was fixed to 0. This model also showed an adequate goodness-of-fit (see Table 3, respecified model). According to the confidence intervals, all of the parameters were significant (*p* < 0.01). The respecified model is represented in Figure 1.

A test of the indirect effects was calculated on the respecified model. We observe that perceived stress seems to mediate the relations between open and problematic communication and verbal CVP, as indicated by the significant indirect effects in both cases (β of the indirect effect and confidence interval are providen): β IND = −0.07 (IC = −0.04, −0.007; *p* < 0.01) and β IND = 0.04 (IC = 0.02, 0.07; *p* < 0.01), respectively. It should be noted that this mediation would be partial in the case of the relation between communication problems and verbal CPV, given the existence of a direct relationship between both variables (β = 0.39).

Lastly, the respecified model was calculated in a second sample obtained with the SPSS. Results showed an adequate goodness-of-fit of the model in the second sample (see Table 3, replicated model). According to the confidence intervals, all the parameters were significant at *p* < 0.01, except the path perceived stress to verbal CPV, which was significant at *p* < 0.10.

## 4. Discussion

This study has tested the fit of a descriptive model between family communication and perceived stress and verbal CPV in adolescents. The results indicate that the positive or negative characteristics of family communication promote or inhibit the stress perceived by the adolescent. This perceived stress was in turn a predictor of verbal CPV. Furthermore, problematic communication between parents and children was also directly related to verbal CPV.

Specifically, in this study, open communication was a protective element against the stress perceived by the adolescent. In other words, adolescents who perceived positive communication within the parent-child dyad, based on freedom, free interchange of information, understanding, and trust, experienced life events as less unpredictable, uncontrollable, and overwhelming. In turn, and as seen in other studies [13,14,25,31,32,33,34,35], perceived stress was a risk factor for verbal CPV (threats, insults, and blackmail toward parents).

In relation to problematic communication (characterized as inefficient, excessively critical, or negative, as well as a resistance to sharing information and its affect), the results showed that this type of communication was both directly and indirectly (through the mediation of perceived stress) related with verbal CPV. The direct relationship may arise in adolescents who perceive that their parents do not listen to them and are excessively critical, resulting in them resorting to verbal violence as an automatic response. In effect, it has been observed that adolescents who believe they matter less to their families will more likely threaten or engage in intrafamilial violence [54]. This result agrees with those of previous studies, which have shown that the deficits in family communication are related with CPV [8,24,25,26,27]. Our results can also be interpreted in the context of the hypothesis that family violence is bidirectional. In accord with this idea, previous studies have shown that the violence of parents towards their children helps to explain a considerable amount of the adolescent CPV [33,55,56]. This hypothesis is in line with an ecological approach to human development. From this point of view, proximal relationships between children and their key social partners in their immediate surroundings (such as parents) are assumed to be bidirectional in nature and explain the “engine” of development and individual differences in behavioral adjustment [10]. In effect, in qualitative studies, it has been shown that conflict between parents and adolescents involves reciprocal exchanges in which family members influence and shape each other’s behavior [57,58]. However, more research is needed to clarify these bidirectional relationships in the genesis and maintenance of the verbal CPV.

The indirect relationship arose from the role of perceived stress in the relation between family communication and verbal CPV. According to these results, it seems that those adolescents who perceived more problems in the communication with their parents perceived greater stress in their lives and also displayed greater amounts of verbal violence toward their parents. Inversely, open communication between parents and adolescents was associated with less perceived stress in adolescents and consequently less verbal CPV. These results point to a mediating role of perceived stress, which would explain the mechanism linking the quality of family communication to verbal CPV.

We can interpret these results from the theoretical framework developed by Olson et al. [11]. In their circumplex model of family functioning, family communication is an important promotor of the quality of the family system as it facilitates cohesion and flexibility. In this way, we can interpret that those adolescents who perceive problems in communication with their parents live in family contexts in which it is difficult to change the norms (low flexibility) and maintain an adequate link between its members (low cohesion). These are characteristics related to an authoritarian parenting style, and, recently, it has been shown that authoritarianism is the parenting style more related to CPV in Spanish adolescents [59]. In addition, these two points (flexibility and cohesion) constitute essential family resources to face the typical changes associated to the adolescent transition [60]. In consequence, verbal CPV can be interpreted as a symptom of a family system ill-equipped to deal with the adolescent transition.

This study has some limitations which are necessary to point out. Firstly, causal relationships could not be established between the variables due to the transversal character of the research. The results obtained point to a possible explanatory mechanism of stress in the relationship between family communication and CPV. Only the availability of longitudinal data collected in future research would allow us to confirm the direction of this relationship between variables. Secondly, the data has been collected solely through self-report. The collection of data from the parents about their communication with their children could be of great use. Despite this, with regard to self-reports in behavioral problems, previous studies have indicated the reliability of self-reports compared with information obtained from parents [61,62]. Finally, there are many factors that can lead to a more complex model of CPV from an ecosystemic approach that are not included in this study. In future research, meso-, exo-, or macrosystemic variables, such as violent family contexts, violent peer interactions, or the permissiveness of corporal punishment within communities among others could be included in the model.

## 5. Conclusions

In conclusion, this study contributes to the current knowledge of CPV from an ecosystemic perspective due to our simultaneous consideration of individual and family variables. The results partly indicate that the characteristics of family communication have an influence on the presence of CPV, given that they affect the levels of perceived stress by the adolescent. Moreover, problematic family communication is directly related to verbal CPV. Hence, two aspects can be outlined regarding the scope of CPV intervention. Firstly, any intervention would need to focus on the family context with the object of promoting open communication among its members, in which ideas and feelings can be freely expressed and communication related problems, such as excessive criticism or negativity, can be reduced. Secondly, there may be a need for a parallel intervention focused on developing adequate stress management strategies in adolescents.

## Figures and Tables

**Table 1:** Pearson correlations among observed variables and descriptive statistics.

Observed Variables	1	2	3	4	5	6	7	8	9	10	11	12	13
**1. CP1**	1												
**2. CP2**	−0.539 **	1											
**3. CP3**	0.125 **	0.236 **	1										
**4. OC1**	−0.350 **	−0.240 **	0.030	1									
**5. OC2**	−0.382 **	−0.265 **	0.056	0.730 **	1								
**6. OC3**	−0.342 **	−0.288 **	−0.003	0.743 **	0.788 **	1							
**7. VCPV1**	0.361 **	0.362 **	−0.032	−0.172 **	−0.224 **	−0.212 **	1						
**8. VCPV2**	0.292 **	0.306 **	−0.065 *	−0.153 **	−0.207 **	−0.187 **	0.518 **	1					
**9. VCPV3**	0.196 **	0.204 **	0.001	−0.122 **	−0.138 **	−0.158 **	0.330 **	0.299 **	1				
**10. PS1**	0.262 **	0.206 **	0.072 *	−0.226 **	−0.235 **	−0.228 **	0.245 **	0.179 **	0.218 **	1			
**11. PS2**	0.181 **	0.171 **	0.017	−0.253 **	−0.223 **	−0.257 **	0.128 **	0.079 **	0.078 **	0.172 **	1		
**12. PS3**	0.185 **	0.128 **	0.009	−0.205 **	−0.202 **	−0.217 **	0.110 **	0.067 *	0.063 *	0.202 **	0.474 **	1	
**13. PS4**	0.273 **	0.267 **	0.107 *	−0.216 **	−0.249 **	−0.222 **	0.240 **	0.186 **	0.173 **	0.569 **	0.189 **	0.210 **	1
**Mean**	1.83	2.34	3.15	3.77	3.83	3.55	1.48	0.460	0.479	1.90	2.03	2.33	2.38
**Standard Deviation**	0.700	0.738	0.708	0.773	0.788	0.870	1.01	0.760	0.772	0.848	0.866	0.871	0.822
**Skewness**	1.15	0.497	−0.093	−0.661	−0.763	−0.383	0.422	2.11	2.17	0.693	0.518	0.163	0.175
**Kurtosis**	1.63	0.516	0.369	0.495	0.634	−0.126	−0.332	4.65	5.07	−0.199	−0.402	−0.554	−0.344

Note: Communication problems (CP); open communication (OC); verbal child-to-parent violence (VCPV); perceived stress (PS); levels of significance: ** *p* < 0.01; * *p* < 0.05.

**Table 2:** Covariances and omega values for each latent factor.

Latent Factor	1	2	3	4	Omega
**1. OC**	0.41				0.96
**2. CP**	−0.17	0.29			0.83
**3. PS**	−0.16	0.17	0.41		0.81
**4. VCPV**	−0.07	0.11	0.09	0.12	0.95

Note: Communication problems (CP); open communication (OC); verbal child-to-parent violence (VCPV); perceived stress (PS).

**Table 3:** Fit indices and path coefficients for structural models.

Model	Fit Indices						R2		Path Coefficient			
	χ2	df	RMSEA	SRMSR	CFI	IFI	PS	VCPV	Relation	Parameter (Standardized Parameter)	95% CI	
			(LL, UL)								LL	UL
Base	7.92	59	0.08	0.06	0.92	0.92	0.28	0.39	CP with OC	−0.17 *** (−0.48)	−0.57	−0.40
			(0.07, 0.08)						CP on PS	0.46 *** (0.39)	0.30	0.47
									CP on VCPV	0.33 *** (0.52)	0.40	0.63
									OC on PS	−0.22 *** (−0.22)	−0.30	−0.12
									OC on VCPV	0.00 (0.01)	−0.09	0.11
									PS on VCPV	0.09 *** (0.17)	0.09	0.26
Respecified	7.79	60	0.07	0.06	0.92	0.92	0.28	0.39	CP with OP	−0.17 *** (−0.48)	−0.57	−0.40
			(0.07, 0.08)						CP on PS	0.46 *** (0.39)	0.30	0.47
									CP on VCPV	0.33 *** (0.52)	0.43	0.60
									OC on PS	−0.22 *** (−0.22)	−0.30	−0.12
									PS on VCPV	0.09 *** (0.17)	0.08	0.26
Replicated	7.36	60	0.07	0.06	0.92	0.92	0.22	0.35	CP with OP	−0.18 *** (−0.48)	−0.54	−0.40
			(0.07, 0.08)						CP on PS	0.49 *** (0.39)	0.30	0.47
									CP on VCPV	0.33 *** (0.55)	0.45	0.62
									OC on PS	−0.14 *** (−0.14)	−0.22	−0.06
									PS on VCPV	0.04 * (0.08)	0.00	0.17

Note: Degrees of freedom (df); root mean square error of approximation (RMSEA); standardized root mean square residual (SRMSR); comparative fit index (CFI); incremental fit index (IFI); confidence interval (CI); lower limit (LL); upper limit (UL); communication problems (CP); open communication (OC); verbal CPV (VCPV); perceived stress (PS); levels of significance: * *p* < 0.10; *** *p* < 0.01.
